# Transcriptomic analysis of humic acid in relieving the inhibitory effect of high nitrogen on soybean nodulation

**DOI:** 10.3389/fpls.2023.1196939

**Published:** 2023-07-26

**Authors:** Wenhua Zhang, Jia Li, Hongya Li, Dongdong Zhang, Baocheng Zhu, Hongli Yuan, Tongguo Gao

**Affiliations:** ^1^ Hebei Engineering Research Center for Resource Utilization of Agricultural Waste, College of Life Sciences, Hebei Agricultural University, Baoding, China; ^2^ State Key Laboratory of Agrobiotechnology and Key Laboratory of Soil Microbiology, Ministry of Agriculture, College of Biological Sciences, China Agricultural University, Beijing, China

**Keywords:** high nitrogen, humic acid, nodulation, relieve inhibition, transcriptome

## Abstract

**Introduction:**

Nitrogen fertilizer intake promotes soybean growth before the formation of nodules, but excess nitrogen has an inhibitory effect on soybean nodulation. It is important to balance nitrogen levels to meet both growth and nodulation needs.

**Methods:**

the nitrogen level suitable for soybean growth and nodulation was studied, the role of humic acid (HA) in alleviating the inhibition of high nitrogen on soybean nodulation was analyzed, and transcriptomic analysis was performed to understand its mechanism.

**Results:**

The results showed that a lower level of nitrogen with 36.4 mg urea per pot could increase the number of nodules of soybean, and a higher level of nitrogen with 145.9 mg urea per pot (U4 group) had the best growth indicators but inhibited nodulation significantly. HA relieved the inhibitory effect at high nitrogen level, and the number of nodules increased by 122.1% when 1.29 g HA was added (H2 group) compared with the U4 group. The transcriptome analysis was subsequently performed on the H2 and U4 groups, showing that there were 2995 differentially expressed genes (DEGs) on the 25th day, accounting for 6.678% of the total annotated genes (44,848) under the test conditions. These DEGs were enriched in mitogen-activated protein kinase signaling pathway-plant, flavonoid biosynthesis, and plant hormone signal transduction based on the –log10 (*P*_adjusted_) value in the Kyoto Encyclopedia of Genes and Genomes pathway (KEGG).

**Discussion:**

HA balanced the nitrogen level through the above pathways in soybean planting to control the number of nodules.

## 1 Introduction

Nitrogen fertilizer application can make up for the lack of nitrogen before nodulation and promote soybean growth. However, excessive nitrogen in the external environment can not only inhibit the number of nodules and nitrogen fixation efficiency of soybean but also cause environmental pollution (Nishida and Suzaki, 2018; Du et al., 2020). Slow-release nitrogen is extremely important for soybean growth and nodulation.

Nitrogen is an indispensable element for plants. About 78% of nitrogen in the atmosphere exists in the form of nitrogen and cannot be directly used by plants. Biological nitrogen fixation can convert nitrogen in the air into ammonia and nitric acid available to plants (Bulen, 1965). The symbiotic nitrogen fixation (SNF) system formed by legumes and rhizobia has the strongest nitrogen fixation ability, which can meet 50%–80% of the total nitrogen required for the growth of legumes, among which the soybean and rhizobia system accounts for 77% of the total symbiotic nitrogen fixation (Herridge et al., 2008).

In soybeans, the nitrogen produced by symbiotic fixation is not always sufficient for achieving high yields (Kunert et al., 2016). When cultured in a nitrogen-free medium, the root nodules appeared as the early senescence type and could not form normal nodules (Banba et al., 2001). Therefore, applying appropriate amounts of nitrogen can supplement the nitrogen required by legumes before nodulation, play a “start-up effect,” and promote the formation of root nodules and their nitrogen fixation capacity (Afza et al., 1987; Sincik et al., 2009). High-nitrogen conditions due to the excessive use of nitrogen fertilizer suppress nodulation and nitrogen fixation, negatively affecting soybean yield. Both nodule formation and nitrogen fixation processes are energetically expensive processes. In the case of enough external nitrogen, a long-distance signaling known as autoregulation of nodulation (AON) should strictly control the number of nodules in host plants and inhibit rhizobia infection (Tiwari et al., 2021; Mengke et al., 2020), root nodule formation and development, and nitrogenase activity (Eaglesham, 1989). This even results in overgrown plants and a fall in productivity and grain quality, creating a disease-prone environment (Petricka et al., 2012; Almeida et al., 2016; Wang et al., 2021). Different form of high-level chemical nitrogen had an inhibitory effect on nodulation of soybean (Yamashita et al., 2019). Urease of soil microorganisms catalyzes the hydrolysis of urea to ammonium, which has a less inhibitory effect on nodulation than nitrate (Paradiso et al., 2015).

Humic acid (HA) is an organic macromolecule produced though chemical or biological decomposition of animal and plant debris and microbial cells (Hayes, 1997). The main constituents of HAs are aromatic rings, aliphatic groups, and other functional groups such as carboxyl group, alcohol hydroxyl group, sulfonic acid group, phenol hydroxyl group, methoxy group, ketone group, enol group, and quinone group (Schulten and Schnitzer, 1993). HA can participate in the transformation of inorganic matter and the mineralization of organic matter, and also promote the absorption, transportation, and distribution of nitrogen by plants and biological nitrogen fixation (Gao et al., 2020). Because of its hydroxyl structure, HA can adsorb bound ammonia, maintain NH_4_^+^ in soil, resist microbial nitrification and denitrification, reduce its volatilization, and regulate soil nitrogen (Burge and Broadbent, 1961; Susilawati et al., 2009). Introducing HA into the interlayer space of bentonite and then combining it with urea released nitrogen slowly, which ultimately led to increased wheat yield and nitrogen uptake (Shen et al., 2020a). Further, HA could also form a complex with urea having phenolic hydroxyl and carboxyl groups to form HA–urea complex, which could alleviate urea hydrolysis. Urea is mainly hydrolyzed into ammonia and carbon dioxide by urease in soil. HA can not only inhibit and stabilize urease activity (Serban and Nissenbaum, 1986) but also alleviate ammonia volatilization after urea hydrolysis. Previous studies have shown that low-molecular-weight HA inhibits urease activity, while high-molecular-weight HA stabilizes urease activity (Marzadori et al., 2000).

There is general consensus that HAs can promote plant growth in an eco-friendly manner. Moreover, HAs, as biostimulants, increase the number of nodules of legumes and improve nitrogen fixation efficiency (Haghighi et al., 2011; Gao et al., 2015). Water-soluble humic materials (WSHM) at a concentration of 500 mg/L significantly promoted nodulation and nitrogen fixation in soybean and increased the biomass, plant height, and root length of soybean, as well as the chlorophyll content. WSHM increased the number of nodules in soybean by 30.5%, the nodule fresh weight by 36%, and the activity of nitrogenase by 30% (Gao et al., 2015). The structure of HA was determined using the pyrolysis gas chromatography–mass spectrometry (py-GC-MS), and flavonoid analogues were observed, which might have a positive effect on soybean nodulation.

HAs can stimulate the growth of legumes and improve the number of nodules and yield under low-nitrogen conditions. However, no studies reported the role of HA on the growth and nodulation of legumes at high-nitrogen level. This study hypothesized that HA alleviated the release of urea and the inhibition of high nitrogen on soybean nodulation under high-nitrogen conditions.

## 2 Materials and methods

### 2.1 HA and strain

HA chosen in this study was the product of *Penicillium* sp. P6 fermentation of lignite, as previously described (Dong and Yuan, 2009), comprising 45.5% HA and 11.7% WSHM. The HA in biodegraded lignite had 2.28% nitrogen, 54.56% carbon, 3.82% hydrogen, and 38.85% oxygen.

*Sinorhizobium fredii* strain CCBAU45436 was provided by Rhizobium Research Center, China Agricultural University, Beijing, China (Zhang et al., 2011). This strain was cultured aerobically at 28°C, 180 r/min in yeast malt broth (Gao et al., 2015).

### 2.2 Effect of urea on soybean nodulation

Urea was used to determine the concentration of nitrogen to study the role of HA in relieving high-nitrogen inhibition of soybean nodulation (The experimental sketch map is shown in **Supplementary figure 1A**).

Soybean seeds were sterilized on the surface through a two-step process involving a 30-second treatment with 95% ethanol followed by a 5-minute treatment with 0.2% HgCl_2_, and then washed six times using sterile water. The seeds were germinated on 0.8% agar-water plates in the dark at 25°C for 24 hours. Once germinated, they were planted in nitrogen-free nutrient solution moisturized vermiculite (Lyu et al., 2019) and 20 mg KH_2_PO_4_ and 1 mL bacterial culture (approximately 10^6^ cells) were added in pots (500 mL). The pots were divided into five parallel treatments, and different amounts of urea were added: U1 (18.2 mg), U2 (36.4 mg), U3 (72.9 mg), U4 (145.9 mg), and U5 (291.8 mg). All the plants were grown under a photoperiod of 16/8-h light/dark cycles at 23°C in a light incubator for 21 days. Three biological replicates were established for each treatment, and 5 plants were randomly selected from each replicate to measure the number, fresh weight, and leghemoglobin content of nodules, as well as the growth index of soybean. The leghemoglobin content was determined as proposed by Becana (Becana et al., 1986) and Riley (Riley and Dilworth, 1985).

### 2.3 Alleviating effect of HA

The effect of HA in terms of relieving the inhibition of high nitrogen on soybean nodulation was examined by the method discussed in Section 2.2. Different amounts of HA were added to potted soybean under high-nitrogen conditions, and the aforementioned high-concentration urea was used as the control group (**Supplementary figure 1B**). Four treatment groups with different amounts of HA were set: H1 (0.43 g HA), H2 (1.29 g HA), H3 (2.16 g HA), and H4 (4.31 g HA). The growth index, number, fresh weight, and leghemoglobin content of nodules were measured. The chlorophyll content in soybean leaves was determined using an SPAD-502 chlorophyll meter (Konica Minolta, Tokyo, Japan).

### 2.4 Urea, ammonium nitrogen, and urease in vermiculite

The residual urea, ammonium nitrogen, and urease activity in vermiculite were determined by diacetyl monoxime colorimetry (Reay et al., 2019), phenol-sodium hypochlorite colorimetry (Kere et al., 2018) and colorimetry (Khan et al., 2020), respectively, to evaluate the hydrolytic ability of urea.

### 2.5 RNA extraction, sequencing, and analysis

After 25 days of cultivation as described previously, the nodules under high-nitrogen conditions treated with and without HA were collected for transcriptomic analysis. The total RNA of nodules was isolated using the TRIzol reagent (Invitrogen, CA, USA) following the manufacturer’s protocol. The quality and integrity of total RNA were examined using the Nanodrop 2000 spectrophotometer (Thermo Fisher Scientific) and the Bioanalyzer 2100 systems (Agilent). The mRNA enrichment, fragmentation into small pieces, cDNA library construction, sequencing, data filtering, and mapping were performed commercially by Meiji Biotechnology Co., Ltd., Shanghai, China. Sequencing was performed on the Illumina HiSeq X Ten platform. After sequencing, Fastp software (0.19.5) was used for quality control of sequencing data. The original paired end reads were pruned and quality-controlled using SeqPrep and Sickle. The quality-controlled Clean Reads were mapped onto the *Glycine_max* genome (reference genome Version: Wm82.a4) using HISAT2 (Version 2.1.0) (http://ccb.jhu.edu/software/hisat2/index.shtml). The mapped reads were spliced using

Cufflinks software (http://cole-trapnell-lab.github.io/cufflinks/). The expression level of each transcript was calculated according to the TPM (the transcripts per million reads) method, false discovery rate (FDR) <0.05 and |log2FC (fold-change) | ≥ 1 were set as the cutoff criteria to identify differentially expressed genes (DEGs) using DESeq2. RNA-seq by expectation maximization (RSEM) was used to quantify gene abundances. (http://cole-trapnell-lab.github.io/cufflinks/) (Trapnell et al., 2010), the mapped reads of each sample were assembled using StringTie (Kim et al., 2015). Function annotation and enrichment analysis of genes were performed based on Gene Ontology (GO, http://www.geneontology.org/) (Young et al., 2010) and Kyoto Encyclopedia of Genes and Genomes (KEGG) Orthology (http://www.genome.jp/kegg/) (Kanehisa et al., 2008). KOBAS software (Mao et al., 2005) was used to test the statistical enrichment of DEGs in the KEGG pathway. All sequencing data are available in the National Center for Biotechnology Information Sequence Read Archive under the accession number PRJNA918980.

### 2.6 Quantitative real-time polymerase chain reaction verification

Total RNA was extracted from nodules using the TRIzol reagent for verifying gene expression (Invitrogen, Carlsbad, CA, USA), and the cDNA was synthesized using the HiScript III RT SuperMix for quantitative polymerase chain reaction (qPCR) (+gDNA wiper) following the manufacturer’s protocols (Vazyme Biotech, Nanjing, China). The Bio-Rad CFX qPCR instrument (Bio-Rad Hercules, CA, USA) was used for quantitative real-time PCR (qRT-PCR), and the data were analyzed using Bio-Rad CFX Manager 3.0 software (Bio-Rad). The expression of target genes was verified using *Actin11* and *CYP2* as internal reference genes (Le et al., 2012).

### 2.7 Statistical analysis

All assays were performed in triplicate, and each experiment was replicated at least three times. The experimental data were presented as the means ± standard deviation (SD). Statistical analyses were carried out using the SPSS Statistics 23.0 software.

## 3 Results

### 3.1 Effect of different urea levels on soybean nodules and growth

**Figure 1:**
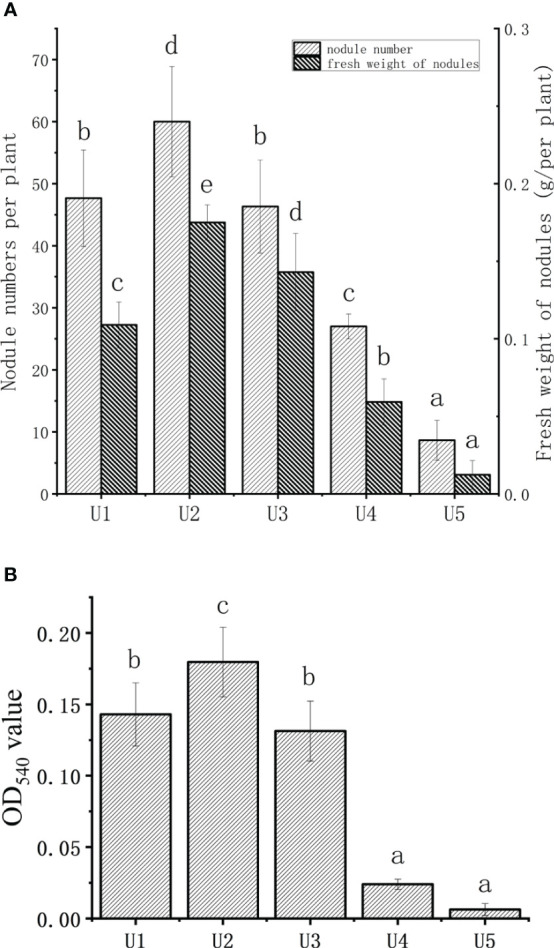
Effect of different urea levels on the number and fresh weight of soybean nodules **(A)**, and leghemoglobin of soybean nodules **(B)**. U1, treated with 18.2 mg urea; U2, treated with 36.4 mg urea; U3, treated with 72.9 mg urea; U4, treated with 145.9 mg urea; U5, treated with 291.8 mg urea. The data were presented as the means ± standard deviation. Different letters indicated significant differences (P < 0.05) among groups.

**Figure 1A** shows the impact of urea dosage on the number of nodules and fresh weight of soybean. The number and fresh weight of soybean nodules initially increased, followed by a decline with increasing concentration of urea. Compared to the U1 group, the U2 group exhibited a 25.9% increase in nodule numbers, while significantly decreasing by 43.4% and 81.1% in the U4 and U5 groups, respectively. The effect of urea dosage on the fresh weight of nodules was consistent with the change in the number of nodules. The fresh weight of nodules in the U2 group increased by 60.5% compared to that of the U1 group, and the fresh weight of root nodules in the U4 and U5 groups was significantly lower than that in the U1 group (*P* < 0.05).

The leghemoglobin content of soybean nodules under different urea levels is shown in **Figure 1B**. The OD_540_ value of the U2 group was 22.6% higher than that of the U1 group, which was also significantly higher than that in the U4 and U5 groups. Both the fresh and dry weights of above-ground exhibited an increasing trend followed by a decreasing trend with the increasing application of urea (**Table 1**). The U4 group outperformed significantly, with an increase of nearly 109% in the above-ground fresh weight and 59% in the above-ground dry weight compared with the U1 group. The U3 group reached the maximum in terms of root fresh and dry weights, exhibiting a 66.7% and 73.3% increase compared to the control group, respectively. The chlorophyll content in leaves increased with the increase in urea dosage. Based on the aforementioned experiments, U4 significantly reduced the number of nodules and improved the growth index compared to the control group. Therefore, the U4 group was considered in this study as the control group with a high level of nitrogen to investigate the potential alleviating effect of HAs on soybean nodulation inhibition caused by high nitrogen.

**Table 1:** Effect of urea dosage on soybean growth index.

	Plant height(cm)	Root length (cm)	Above ground fresh weight (g)	Root fresh weight (g)	Aboveground dry weight (g)	Root dry weight (g)	Chlorophyll (SPAD value)
U1	28.87 ± 0.32a	19.50 ± 0.67a	1.94 ± 0.06a	0.91 ± 0.06a	0.32 ± 0.03a	0.08 ± 0.006a	25.35 ± 3.84a
U2	31.83 ± 1.74a	24.37 ± 1.00c	3.09 ± 0.16b	1.92 ± 0.07b	0.60 ± 0.05b	0.23 ± 0.001c	30.70 ± 3.76b
U3	29.23 ± 1.29a	21.73 ± 0.50b	3.68 ± 0.14bc	2.73 ± 0.03c	0.76 ± 0.02c	0.30 ± 0.009d	35.75 ± 2.73c
U4	40.10 ± 1.80b	22.78 ± 0.87bc	4.06 ± 0.21c	2.08 ± 0.22b	0.78 ± 0.03c	0.22 ± 0.021c	35.00 ± 1.71c
U5	28.33 ± 2.50a	23.68 ± 0.34bc	3.85 ± 0.28c	1.77 ± 0.21b	0.71 ± 0.02c	0.15 ± 0.019b	40.00 ± 3.20d

U1: Treated with 18.2 mg urea; U2: Treated with 36.4 mg urea; U3: Treated with 72.9 mg urea; U4: Treated with 145.9 mg urea; U5: Treated with 291.8 mg urea. The means followed by different letters were significantly different at P < 0.05, which was indicated by the means ± standard deviation (n = 3).

### 3.2 HA relieved the inhibition of high nitrogen on soybean nodulation

**Figure 2:**
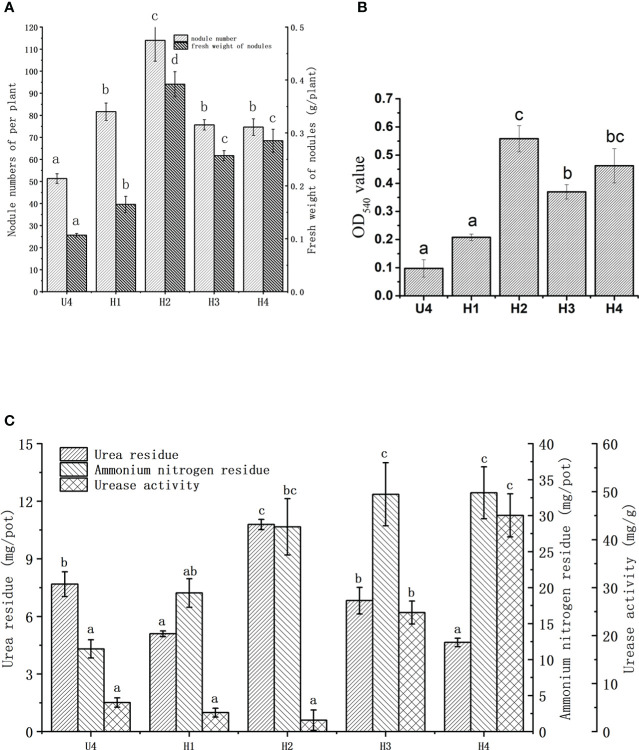
Effects of HA on the number **(A)** and fresh weight **(B)** of root nodules, urea residues, ammonium nitrogen residues, and urease activity in vermiculite **(C)** under the high level of nitrogen. U4, the control group; H1, treated with 0.43 g HA; H2, treated with 1.29 g HA; H3, treated with 2.16 g HA; H4, treated with 4.31 g HA. The data were presented as the means ± standard deviation. Different letters indicated significant differences (P < 0.05) among groups.

**Figure 2A** illustrates the alleviating effect of HA on soybean nodulation inhibition under a high level of nitrogen. The number of nodules in the four treatment groups (H1, H2, H3, and H4) with HA significantly increased by 59.1%, 122.1%, 47.4%, and 45.5%, respectively, compared with that in the control group (U4 group) treated with urea only. The number of nodules reached the maximum value of 114.0 in the H2 group (1.29 g HA added).

The treatment with HA increased the fresh weight of nodules compared with that in the control group (**Figure 2A**); especially, the fresh weight in the H2 group reached the maximum value of 0.39 g, representing a significant rise of 267.5% compared to the control group. The fresh weight of root nodules in other HA treatment groups also exhibited significantly increases compared to the control group. In general, the change pattern in the number of soybean nodules and fresh weight was consistent.

The leghemoglobin content of nodules increased on adding HA. The most significant increase was observed in the H2 group with an OD_540_ value of 0.558, followed by that in the H4 and H3 groups. HA had a relieving effect on the leghemoglobin content after urea treatment (**Figure 2B**). At the same time, in order to ensure meaningful leghemoglobin content, the nitrogenase activity was tested (**Supplementary figure 2**). The results of this part prove that soybean leghemoglobin content is valuable, indicating that the nodules had nitrogen fixation activity. The influence of HA on the soybean growth index under a high level of nitrogen is depicted in **Table 2**. The addition of HA did not change the chlorophyll content of the plants under a high level of nitrogen. The root length, above-ground fresh weight, and root fresh weight of soybean increased significantly in the H2 group compared with the control group. Among these, the H2 group demonstrated a 28.14% increase in above-ground fresh weight and a 57.8% increase in root fresh weight compared to the control group, respectively. The plant height in the H3 and H4 groups showed significant differences compared with that in the control group, which increased by 41.4% and 39.2%, respectively.

**Table 2:** Effect of HA on soybean growth index under a high level of nitrogen.

	Chlorophyll (SPAD value)	Plant height (cm)	Root length (cm)	Aboveground fresh weight (g)	Root fresh Weight (g)
U4	50.27 ± 0.71ab	29.07 ± 3.47a	23.67 ± 0.43a	3.02 ± 0.11a	2.25 ± 0.14a
H1	48.50 ± 0.49a	35.40 ± 3.23ab	26.33 ± 0.54ab	3.35 ± 0.12ab	2.93 ± 0.09b
H2	51.93 ± 0.28b	34.53 ± 2.26ab	28.17 ± 1.92b	3.87 ± 0.07d	3.55 ± 0.21c
H3	50.83 ± 1.49ab	41.10 ± 2.40b	27.53 ± 1.77ab	3.45 ± 0.14bc	2.82 ± 0.19ab
H4	51.23 ± 1.39ab	40.47 ± 1.95b	28.47 ± 1.25b	3.82 ± 0.16cd	3.72 ± 0.29c

U4: the control group; H1: treated with 0.43 g HA; H2: treated with 1.29 g HA; H3: treated with 2.16 g HA; H4: treated with 4.31 g HA. The means followed by different letters were significantly different at P < 0.05, which was indicated by the means ± standard deviation (n = 3).

### 3.3 Relationship between HAs and hydrolyzed urea

Urea is decomposed by urease to form ammonia. The urea residue, urease activity, and ammonia nitrogen content in vermiculite were determined to investigate the role of HA in this chemical process (**Figure 2C**). Proper amounts of HA could slow down the hydrolysis of urea. Among all the treatment groups, the H2 group showed a significantly higher urea residue content of up to 10.79 mg/pot compared to others. The urea residues in the H1 and H4 groups were 33.7% and 39.45% lower than those in the control group, respectively, and there was no significant difference between the H3 group and the control group. The HA in the H2 group exhibited a slow-release effect on urea. The residual ammonium nitrogen level increased with the increase in HA, and was higher in all HA groups compared to the control groups. The level of residual ammonium nitrogen in the H1, H2, H3, and H4 groups was 67.6%, 147.7%, 187%, and 189% higher than that in the U4 group, respectively. Although not significant, the urease activity in the H1 and H2 groups was lower than that in the control group. The urease activity in the H3 and H4 groups was 186.9% and 188.9% higher than that in the control group, respectively.

### 3.4 Transcriptomic analysis of HA in relieving the inhibition of high nitrogen on nodulation

**Figure 3:**
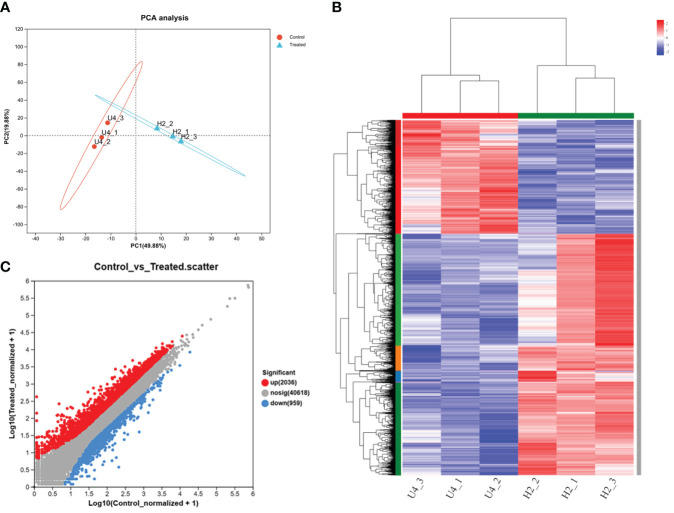
PCA **(A)**, Pearson correlation coefficient analysis of all expressed genes **(B)**, and differential fold of gene expression **(C)** in the treatment groups with and without HA at the high nitrogen level. Numbers within parentheses indicated the percentage of variance explained by each component. Each dot in the graph represents a specific gene: red dots indicate significantly upregulated genes, blue dots indicate significantly downregulated genes, and gray dots indicate non-significantly different genes. All treatments included three parallel samples. Control referred to the U4 group with the high nitrogen level, and treated referred to the H2 group treated with HA at the high nitrogen level on day 25.

A comparative transcriptomic analysis of soybean nodules was performed with and without HA at a high level of urea to reveal the candidate genes involved in HA, relieving the inhibition of high urea content. RNA paired-end sequencing was carried out using the Illumina Solexa platform, resulting in a high proportion of high-quality reads (approximately 93%). The heat map of the correlation between samples verified the scientific validity of biological replicates (**Figures 3A, B**). Upon trimming the raw data, a total of 43,739,118 clean reads were obtained for the U4 sample and 46,537,918.67 clean reads were obtained for the H2 sample (**Table S1**). All clean reads were then mapped to the soybean reference genome, with an average of 84.46% of the reads being uniquely mapped.

**Figure 4:**
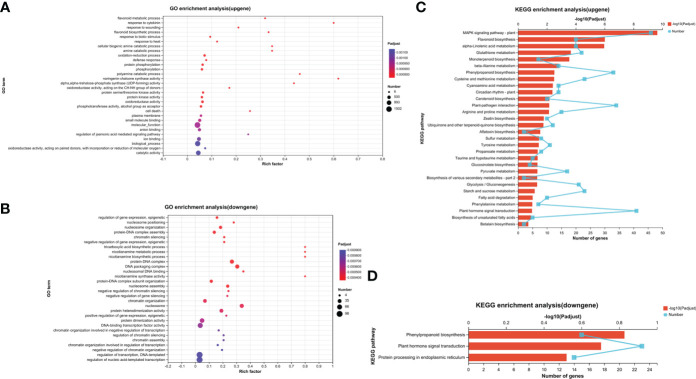
GO enrichment analysis of upregulated genes **(A)** and downregulated genes **(B)**, and KEGG enrichment analysis of upregulated genes **(C)** and downregulated genes **(D)**. Each GO term is represented by a dot, the size of which indicates the number of genes/transcripts in this GO term, and the color of the dot corresponds to a different *P*_adjusted_ range. Different points on the line indicate the number of genes/transcripts in the pathway, and the higher the –log10 (*P*_adjusted_) value, the more significantly the KEGG pathway was enriched. Only the top 30 enrichment results were shown by *P*_adjusted_ < 0.5.

The assay results were screened using differential significance criteria, with a two-fold difference as the threshold for identifying differentially expressed genes (DEGs) with FDR ≤ 0.05. A total of 2995 DEGs were identified, which accounted for 5.66% of the total annotated genes (44,848). Among these DEGs identified, 2036 genes were found to be upregulated while 959 genes were downregulated after adding HA (**Figure 3C**). GO functional annotation analysis of DEGs revealed 7 items associated with biological process, 7 items with cellular component and 6 items with molecular function (**Supplementary figure 3**). The majority of biological processes were comprised of DEGs that participate in metabolic process, cellular process and biological regulation. The cell part, membrane part and organelle part account for the majority of cellular component. In the terms of molecular function, the significant proportion of DEGs were associated with binding, catalytic activity and transporter activity. Only the top 30 enriched GO terms were displayed in upregulated and downregulated genes. The smaller the *P*_adjusted_ value (<0.5), the more significant it became. The most significant term in the upregulated genes’ GO terms was “flavonoid metabolic process,” followed by “response to cytokinin” and “response to wounding” (**Figure 4A**). Increasing the Rich factor leads to a corresponding increase in the degree of enrichment. “Response to cytokinin,” “naringenin-chalcone synthase activity,” and “polyamine catabolic process” were the top three enrichment terms with richness factor greater than 0.4 in the upregulated genes. In the downregulated genes, “tricarboxylic acid biosynthetic process,” “nicotianamine metabolic process,” “nicotianamine biosynthetic process,” and “nicotianamine synthase activity” were the terms with the same Rich factor (0.8) at the top enrichment degree (**Figure 4B**). These 30 terms were significantly upregulated or downregulated (*P*_adjusted_ ≤ 0.05).

The KEGG database showed a significant enrichment of the “mitogen-activated protein kinase (MAPK) signaling pathway–plant,” positively regulating nodule organogenesis in *Lotus japonicus* (Chen et al., 2012), which was enriched with 46 genes (**Figure 4C**), followed by the pathway “flavonoid biosynthesis” with 20 genes and “alpha-linolenic acid metabolism” with 20 genes [–log10 (*P*_adjusted_) > 5]. The three most significantly enriched gene/transcript pathways were “MAPK signaling pathway–plant,” “plant hormone signal transduction” with 41 enriched genes, and “plant–pathogen interaction” with 34 enriched genes. Among these, “zeatin biosynthesis” was significantly upregulated with HA treatment (*P*_adjusted_ = 0.016503162), which is a type of cytokinin that plays a central role in nodule development (Lin et al., 2020). There were 33 enriched genes in the phenylpropanoid pathway, which is responsible for the synthesis of flavonoids as secondary metabolites. Further, the metabolism of some amino acids, such as cysteine and methionine, tyrosine, and propanoate, was significantly upregulated (*P*_adjusted_ < 0.05). Plant–pathogen interaction terms “phenylpropanoid biosynthesis,” “plant hormone signal transduction,” and “protein processing in endoplasmic reticulum” were significantly enriched in the downregulated KEGG pathway (*P*_adjusted_ < 0.5) (**Figure 4D**). The results indicated that HA-triggered mode of action and alterations regulated the expression levels of genes involved in hormone metabolism and signal activity in soybean, and the transcriptome results were consistent with those of root nodules produced on adding HA slow-release nitrogen fertilizer.

### 3.5 RT- PCR verification

**Figure 5:**
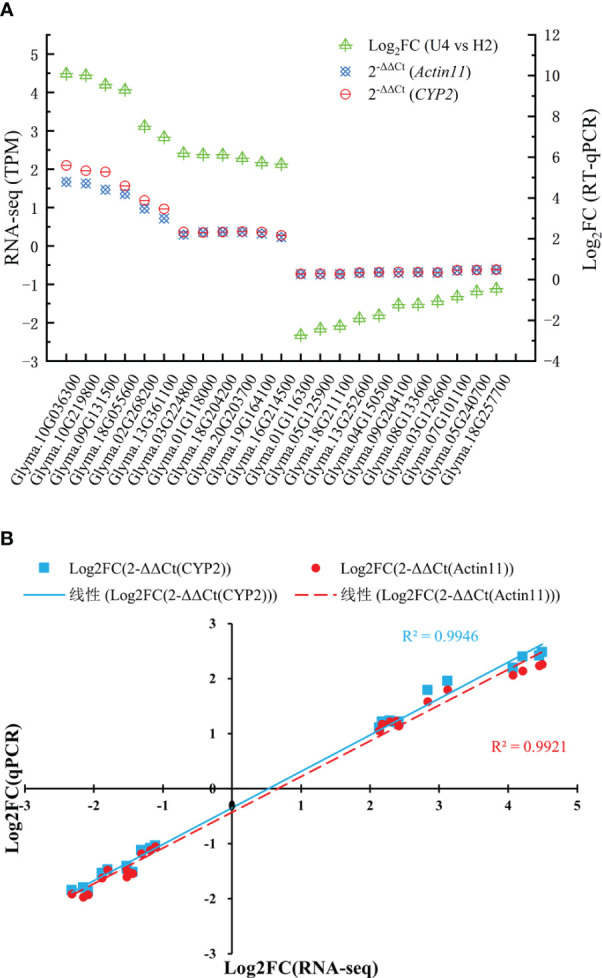
qRT-PCR results showing the mRNA expression level of 23 genes **(A)** and the linear regression **(B)**. Expression levels were normalized to *Actin11* and *CYP2* and were presented relative to controls, which were calculated using 2^-△△Ct^ (mean ± standard deviation).

To validate the expression analysis of RNA sequencing data, 23 key DEGs were selected for qPCR. Of 23 DEGs (**Table 3**), 5 DEGs (*Glyma.10G036300*, *Glyma.19G164100*, *Glyma.20G203700*, *Glyma.08G133600*, and *Glyma.09G204100*) were involved in MAPK signaling pathway–plant, 4 DEGs (*Glyma.09G131500*, *Glyma.16G214500*, *Glyma.07G101100*, and *Glyma.13G252600*) were involved in plant–pathogen interaction, 4 DEGs (*Glyma.10G219800*, *Glyma.18G055600*, *Glyma.05G125900*, and *Glyma.18G211100*) were involved in phenylpropanoid biosynthesis, 2 DEGs (*Glyma.02G268200* and *Glyma.05G2407*) were involved in cysteine and methionine metabolism, 4 DEGs (*Glyma.01G118000*, *Glyma.18G204200*, *Glyma.01G116300*, and *Glyma.18G257700*) were involved in glycolysis/gluconeogenesis, and 4 DEGs (*Glyma.13G361100*, *Glyma.03G224800*, *Glyma.03G128600*, and *Glyma.04G150500*) were involved in plant hormone signal transduction. **Figure 5** displays the qPCR results. The correlation analysis indicated high consistency between qPCR and RNA sequencing data (R^2^_(_ *_CYP2_*_)_ = 0.9946, R^2^_(_ *_Actin11_*_)_ = 0.9921), which demonstrated the reliability of the data.

**Table 3:** Annotation of the 23 validated genes.

Gene name	Primer for Q-PCR	Log_2_FC (Treated/Control)	Regulate	Pathway definition
*Glyma.10G036300*	F: CTCCACAGCACCAACAACAGAAG R: CAGTGTCAAAGGTTCCAAGCCAAA	4.491097	up	MAPK signaling pathway - plant; Plant hormone signal transduction
*Glyma.19G164100*	F: GTCACTACCGTTGCTGCCTTCG R: TGCGGCTTCGGCTGTGTCAA	2.172373	up	MAPK signaling pathway - plant; Plant hormone signal transduction
*Glyma.20G203700*	F: TTCTCCTCCGAATCTTCCTCTCCG R: CGCACACCTCTGTACGACTTCTTC	2.286828	up	MAPK signaling pathway - plant; Plant hormone signal transduction
*Glyma.08G133600*	F: ATTCAGTGAGGACGAGGCGAGAT R: CGTGGTGCTGAGCTTCCATCTAG	-1.518029	down	MAPK signaling pathway - plant; Plant hormone signal transduction
*Glyma.09G204100*	F: ACACCGTCGTCCTCGAATCCTAC R: ACCGTCGCCGTTGGTTCCTT	-1.525996	down	MAPK signaling pathway - plant; Plant hormone signal transduction
*Glyma.13G361100*	F: TACCGCAAGAAGAACACCGTCAA R: TCAGCATTCTCCGCATCCTTCAA	2.834028	up	Plant hormone signal transduction
*Glyma.03G224800*	F: CCCGCTAGTTCTTCCTCTTCCTCT R: ATGCCGAGTCCAAGCCTGAGAT	2.417607	up	Plant hormone signal transduction
*Glyma.03G128600*	F: CAGTAGCAGCAGCGGCAACAAC R: CTCTCGTCCTCAGCGTTATCCAGT	-1.438246	down	Plant hormone signal transduction
*Glyma.04G150500*	F: GAGCCGCTGGATAAGTGGAGGAAG R: AAGCCGAAGCCGCAATGAGAGG	-1.803954	down	Plant hormone signal transduction
*Glyma.09G131500*	F: TGAGGATGTTGACGAGGAGAAGG R: CCAGTCATTGGTGAGGCTCTTGT	4.206525	up	Plant-pathogen interaction; Protein processing in endoplasmic reticulum
*Glyma.16G214500*	F: ATGTTGCCGATCTGCCTGTTGAA R: CCTCCACCTCTCCAGCAGTTGT	2.134464	up	Plant-pathogen interaction; Phosphatidylinositol signaling system; MAPK signaling pathway - plant
*Glyma.07G101100*	F: TTCAACCGCTTCGACGCCAAC R: GCGAACTCTGTGAGGCTGATGAAG	-1.314561	down	Plant-pathogen interaction
*Glyma.13G252600*	F: CCAGACTGCAACCTCGAACACT R: CAGGCACTCATCATGGACACAAG	-1.884467	down	MAPK signaling pathway - plant; Plant-pathogen interaction; Plant hormone signal transduction
*Glyma.10G219800*	F: TGACATTGTAGCAGTAGCAGCAC R: ATCGTCTGTGAATCGTGGAAGGT	4.451012	up	Phenylpropanoid biosynthesis
*Glyma.18G055600*	F: ATCCTTGCTCCGTCTTCATTTCCA R: GGACAAGCACTCTCCAACTGACTT	4.067622	up	Phenylpropanoid biosynthesis
*Glyma.05G125900*	F: GTGCTGACAATGTTCGAGATGCTA R: TGCCTCCACCACCTCCTCTTAT	-2.154422	down	Phenylpropanoid biosynthesis
*Glyma.18G211100*	F: TGCTCTTGGTGGACCTAGTTGGA R: TTTCTGCGGTGGTGAGACCTTTG	-2.083624	down	Phenylpropanoid biosynthesis
*Glyma.02G268200*	F: AGATTGAAGACGCTTGCCAGAACT R: CGACTGCCTCCTTGAACCTGTT	3.120259	up	Cysteine and methionine metabolism
Gene name	Primer for Q-PCR	Log_2_FC (Treated/Control)	Regulate	Pathway definition
*Glyma.05G240700*	F: CATCGCCGTCAATCGTGGAAC R: ATGGTGGAGCAATTAGGGTTAGCA	-1.181538	down	Cysteine and methionine metabolism; Lysine biosynthesis; Glycine, serine and threonine metabolism; Monobactam biosynthesis
*Glyma.01G118000*	F: CAGTGGAAGCAGCAGCAGAGTT R: ATGGCATCACAGCAAATGGATAGC	2.385361	up	Glycolysis/Gluconeogenesis
*Glyma.18G204200*	F: CGGAGCGTACAGCGAGAACTT R: ACAGCCTGGAAGCAAGTAATGGT	2.377114	up	Glycolysis/Gluconeogenesis
*Glyma.01G116300*	F: ATGGCGATGGCGACCTCAATC R: ACAGAGAGACCCACAGCACCTAA	-2.319937	down	Glycolysis/Gluconeogenesis; Fatty acid degradation; Tyrosine metabolism
*Glyma.18G257700*	F: CGGGTTGCTAACAGAATTGGAGGA R: CTGAAGGTAATGCTGGGACTTGGA	-1.113815	down	Glycolysis/Gluconeogenesis; Galactose metabolism

## 4 Discussion

### 4.1 HA inhibited urease activity

Exposure of nodulated roots to high concentrations of combined nitrogen, known as the nitrogen inhibitory effect, represses the development of root nodules and nitrogen fixation activity, which acts *via* mechanisms that are largely unknown (Gibson and Harper, 1985; Xu et al., 2021). In comparison to soybean that didn’t receive nitrogen, the addition of nitrogen led to a decrease of 19% and 52% in below-ground biomass and number of nodules, respectively, across soils. Among all factors influencing root growth parameters, the nitrogen rate was found to be the most critical one (Namvar et al., 2011; McCoy et al., 2018). Lyu et al. (Lyu et al., 2019) used a dual-root soybean system to investigate the impact of nitrogen application on root nodule growth, nitrogenase activity, and nitrogen accumulation, which indicated that high nitrogen application led to a contact-dependent local inhibition of these parameters. The specific nitrogenase activity was systematically regulated throughout the whole plant, and high levels of nitrogen inhibition were found to be recoverable.

Urea fertilizers are extensively utilized due to their ability to significantly increase soil nitrogen levels (Saeed et al., 2017). The SPAD meter is a prevalent tool for non-destructive and efficient measurement of leaf chlorophyll concentrations (Dong et al., 2019). The SPAD value is related to various factors, among which nitrogen is one of the most important factors. SPAD value was positively correlated with nitrogen uptake by plants (Xiong et al., 2015; Yuan et al., 2016). In this study, chlorophyll content increases with the use of urea (**Table 1**), which was consistent with the above conclusion. Humic acid has positive effect on the growth of plant in **Table 2**, which was verified in many reports (Arancon et al., 2006; Maji et al., 2016; Ampong et al., 2022). Urease inhibitors existed in the fertilizers, ensuring the long-term nitrogen release and improvement in nitrogen uptake by plants and nitrogen storage in seeds and silage (Folina et al., 2021). Lignin was an excellent carrier of nutrients for slow-release fertilizers, adsorbing and encapsulating nutrients to achieve the slow-release property (Mulder et al., 2011).

HA formed a complex through carboxyl and phenolic hydroxyl groups. The HA–urea complex could slow down the decomposition of urea and play a slow-release role in urea. Low-molecular-weight HA inhibited the urease activity, and high-molecular-weight HA stabilized the urease activity (Marzadori et al., 2000). However, the urease activity increased with the increase in the ratio between HA and urease (Liu et al., 2019). The hydroxyl group in HA adsorbed and bound ammonia to maintain NH_4_^+^ in soil and reduced ammonia volatilization (Susilawati et al., 2009). Moreover, the greater the amount of HA added to urea fertilizer, the stronger its ability becomes to inhibit ammonia volatilization (Mohd et al., 2009; Shen et al., 2020b). In this study, urease activity was the lowest in the H2 group and the highest in the H3 and H4 groups, which was consistent with the result of urea residue. The amount of residual ammonium nitrogen also increased with an increase in the amount of HA.

### 4.2 HA promoted nodulation by regulating initial nodulation signal transduction

Many studies confirmed that HA could increase the number of nodules of leguminous plants (Capstaff et al., 2020; da Silva et al., 2021). However, no reports explored the effect of HA on the nodulation ability of soybean under high-nitrogen conditions. HA relieved the inhibition of nodulation under high-nitrogen conditions, thereby increasing the number of soybean nodules. Transcriptomics was used to explain this phenomenon. According to KEGG analysis, genes related to the MAPK signaling pathway, flavonoid biosynthesis, α-linolenic acid metabolism, and so on were upregulated, while genes related to plant hormone signal transduction and protein processing in endoplasmic reticulum were downregulated. MAPK cascades acted as a signal transduction pathway, including hormone responses. Yin et al. (Yin et al., 2019) discovered SIP2, an MAP kinase kinase, which interacts with SymRK to promote nodule organogenesis in *L. japonicus*. This suggests that an MAPK cascade might be involved in *Rhizoium*–legume symbiosis. MAPKs play significant roles in nodulation; for instance, MAPKK4 is a positive regulator of nodule formation (Chen et al., 2012), while the pathway involving MKK5 and MPK3/MPK6 negatively regulates the transcription factors NSP1 and ERN1 to inhibit the initial stages of nodule formation (Komis et al., 2018). The activation of the MKK5–MPK3/MPK6–NSP1/ERN1 pathways controls the formation of symbiotic root nodules in *Medicago truncatula* (Ryu et al., 2017). Lee et al. (Lee et al., 2008) prepared genistein induced culture filtrate (GCF) of *Bradyrhizobium japonicum* by Nod factor to induce the early reaction of soybean root hair. It was found that GCF could induce root hair deformation, so the antibody against *GMK1* (*Glycine max* MAP kinase 1) was used to verify that GMK1 was the activated kinase after GCF-treated. The results indicated that MAPK plays a role in establishment of symbiosis between soybean and *B. japonicum*.

### 4.3 HA affected cytokinin signaling in early soybean nodulation

The beneficial role of cytokinins in the initiation of nodule organogenesis has been demonstrated. Nod factors produced by rhizobia induce nodule formation in legumes. This signaling cascade leads to activation of cytokinin signaling and an increase in cytokinin concentration at the nodule initiation site in *Medicago truncatula* (van Zeijl et al., 2015). Although rhizobia can secrete bioactive cytokinins, it cannot replace the role of Nod factors (Podlešáková et al., 2013). In the absence of rhizobia infection, nodule-like structures were found in the roots of chickpea seedlings exogenously treated with appropriate concentration of cytokinin, indicating that cytokinin was sufficient to induce nodular organs (Tiwari et al., 2022). Cytokinin signaling involves regulating the expression of cytokinin primary response genes through type-B response regulator (RRB). Sovanna et al. significantly reduced the number of nodules formed by RNA interference or mutation of *MtRRB3*, which is the RRB-encoding gene most strongly expressed in *M. truncatula* roots and nodules (Tan et al., 2020). Among the various phytohormones that govern cell cycle checkpoints, cytokinins play the most crucial role in limiting cell proliferation at the meristems (Wong et al., 2020). Guinel et al. (Guinel, 2015) clearly illustrated the role of ethylene in nodule organogenesis, functioning, and senescence. The findings demonstrate that ethylene is a crucial component at the center of this highly effective symbiotic relationship. It was reported that nodulation could be affected by the application of ethylene to rhizobia-inoculated root cultures of beans. The results showed a significant reduction, not only in the number of nodules formed but also the amount of fixed nitrogen (Grobbelaar et al., 1971). Pizzeghello et al. highlighted that humic substances (HS) contain a physiologically active concentrations of the cytokinin isopentenyladenosine (IPA), which can enhance plant metabolism (Pizzeghello et al., 2013). Ng et al. investigated the failure to initiate nodulation in the cytokinin perception mutant *cre1* of *M. truncatula*, and proposed that cytokinin signaling mediated CRE1 is essential in regulating flavonoid accumulation, altering auxin transport and auxin accumulation required for nodule initiation in *M. truncatula* (Ng et al., 2015). In addition, cytokinin biosynthesis in shoots may participate in AON system to regulate nodule organogenesis (Gamas et al., 2017). Therefore, it is assumed that humic acid affects the autoregulation of nodulation system and changes the nodulation situation by changing the transport and content of cytokinin in long distances between shoot and root, as observed by Mora et al. ‘s application of humic acid to cucumber (Mora et al., 2010). But so far, there are no reports on the relationship between HA and AON pathways.

### 4.4 HA affected the secondary metabolism of soybean

The transcriptome KEGG enrichment results in this study indicated that HA upregulated the phenylpropane biosynthesis pathway. Flavonoids, the secondary metabolites of the phenylpropanoid pathway, have been identified as key players in the establishment of legume root nodule symbiosis. Flavonoids act as signal molecules that trigger rhizobial nodulation initiation signals and act as inhibitors of polar auxin transport in nodule organogenesis (Gifford et al., 2018). Cinnamic acid 4-hydroxylase, a major rate-limiting component in the biosynthesis of phenanthrene, was significantly differentially expressed in *M. sativa* (Gifford et al., 2018). Gene-specific qRT-PCR was used to quantify the expression of isoflavone synthesis genes in soybean (*Glycine max* L). Chalcone synthase 7, chalcone synthase 8, and isoflavone synthase 1 displayed high basal expression levels in roots compared with hypocotyls. Hence, these genes could be responsible for encoding the isoenzymes that play a major role in the principal substrate flux toward specific isoflavone synthesis in roots (Pregelj et al., 2010). However, this class of genes also showed a significant upregulation trend in HA-treated root nodules, indicating that HA promoted the phenylpropane metabolic pathway, stimulated the production of flavonoids, and enhanced signal expression. The activation of *nod* genes in rhizobia is attributed to daidzein and genistein (Bosse et al., 2021). HA has been considered as a candidate for the auxin effect because it can induce certain enzyme-encoding genes associated with secondary nutrient transport proteins (such as nitrate transporter) (Quaggiotti et al., 2004). LjSWEET3, a member of the SWEET transporter family, shows a significant increase during the development of root nodules in *L. japonicus* and has the highest expression level in mature nodules (Sugiyama et al., 2017). In a high-urea environment, humic acid was found to upregulate the expression of sugar transporter genes (*Glyma.08G010000*; *Glyma.03G149267*), suggesting that humic acid can promote nodulation and provide a source of carbon when soybean is inhibited by high concentrations of urea. Application of fulvic acid resulted in an increase in the number of pink nodules without affecting the nutrient composition (Capstaff et al., 2020). Roomi et al. treated *Arabidopsis* roots with HA and performed proteomic analysis (Roomi et al., 2018). The results of functional annotation analysis indicated that the main molecular functions are binding of copper and manganese ions, nutrient reservoir activity, and protease binding. These findings suggest that HA can stimulate plant energy metabolism and protein synthesis, and may have an impact on metabolic pathways and physiological processes in plants (Roomi et al., 2018). In this study, 6 genes (*Glyma.19G058900*; *Glyma.06G153900*; *Glyma.20G110100*; *Glyma.15G042100*; *Glyma.17G049800*; *Glyma.15G176900*) related to “nutrient reservoir activity” were annotated by GO, but how HA affects the nodulation process of legumes remains though transport pathway is not clear. All the aforementioned findings supported the results of the gene annotation of the transcriptome in this study. HS established transcriptional interactions with biochemical components and signaling pathways, eliciting dynamic signaling within the plant to regulate nodulation.

## 5 Conclusions

High concentrations of urea inhibited soybean nodulation, and HA alleviated the inhibitory effect on soybean nodulation and nitrogen fixation. Following transcriptomic analysis and validation, a more than two-fold variation in the expression levels of “response to cytokinin,” “MAPK signaling pathway-plant,” “flavonoid biosynthesis,” “plant hormone signal transduction,” and “phenylpropanoid biosynthesis” was observed. This demonstrates that HA can modify the cytokinin signal of soybean nodules and regulate the phenylpropyl pathway and MAPK signaling pathway under conditions of high nitrogen to maintain the normal level of nodulation and restores the nitrogen-fixing activity of soybean nodulation. Humic acid can alleviate the inhibitory effects of high levels of nitrogen on soybean nodulation.

## Data availability statement

The datasets presented in this study can be found in online repositories. The names of the repository/repositories and accession number(s) can be found in the article/**Supplementary Material**.

## Author contributions

This paper was co-written and analyzed by WZ and JL. TG took the lead in designing, researching and writing the paper. HL and DZ proofread all drafts. BZ gave preliminary guidance. HY conducted objective review of the paper. All authors have contributed to further revisions of this paper.

## Supplementary material

Supplementary Figure 1 A flow chart with pictures describing the experimental design.

Supplementary Figure 2 Nitrogenase activity of nodules on adding HA. Acetylene reduction assay was used to detect nitrogenase activity.

Supplementary Figure 3 GO functional annotation analysis of DEGs.
