# Supplementary figures and images for: Transcriptomic analysis of humic acid in relieving the inhibitory effect of high nitrogen on soybean nodulation
Source: Front Plant Sci. 2023 Jul 26;14:1196939. doi: 10.3389/fpls.2023.1196939 (PMC10410467; doi:10.3389/fpls.2023.1196939)

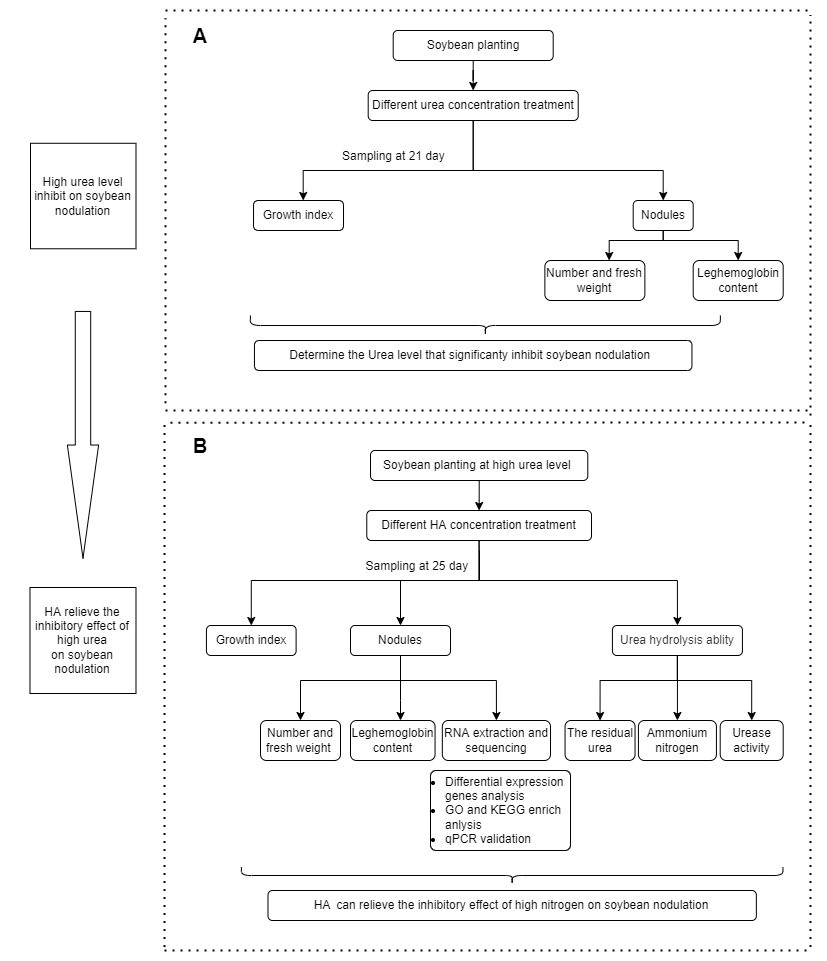

Supplement: Supplementary Figure 1 — A flow chart with pictures describing the experimental design. [file Image_1.jpeg]

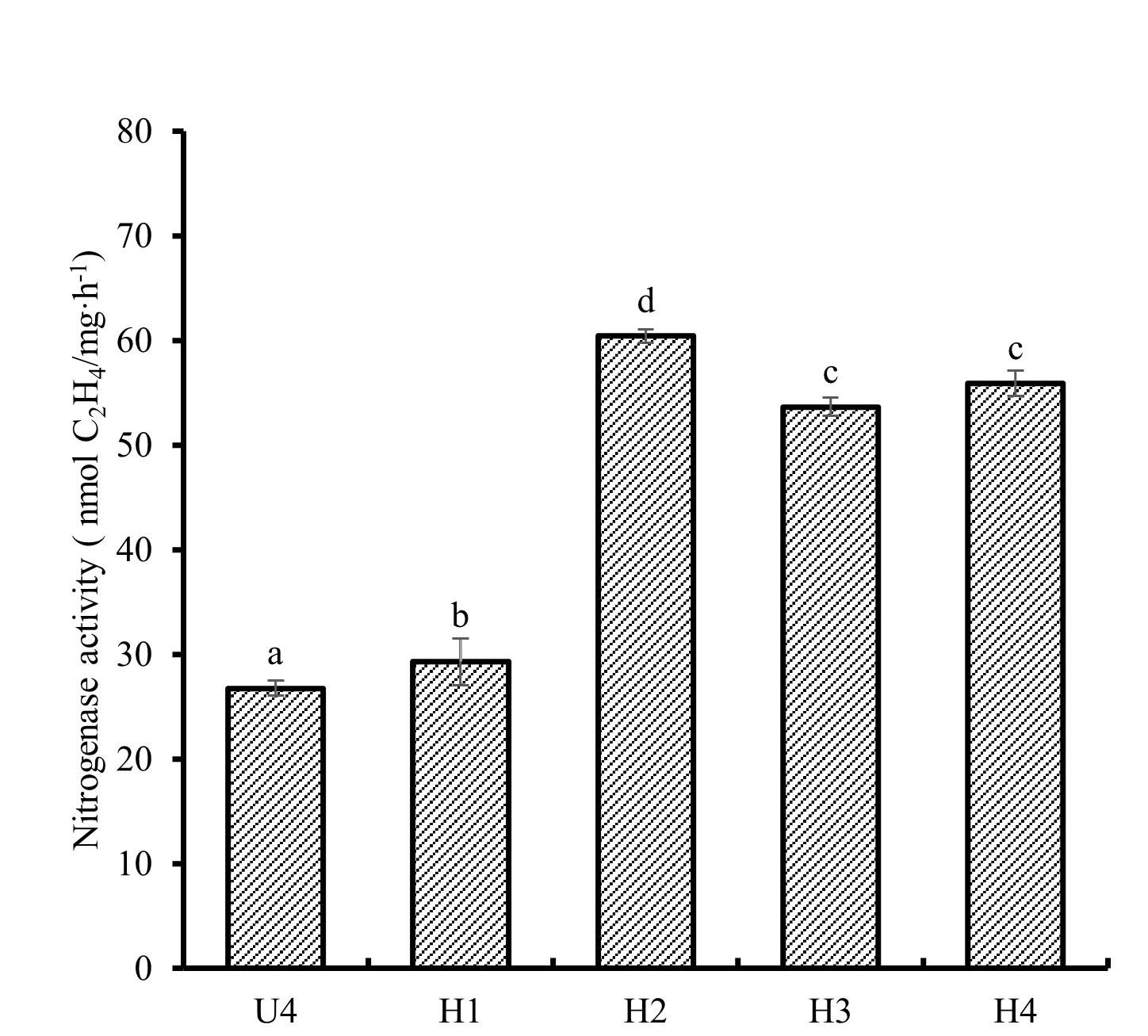

Supplement: Supplementary Figure 2 — Nitrogenase activity of nodules on adding HA. Acetylene reduction assay was used to detect nitrogenase activity. [file Image_2.tif]

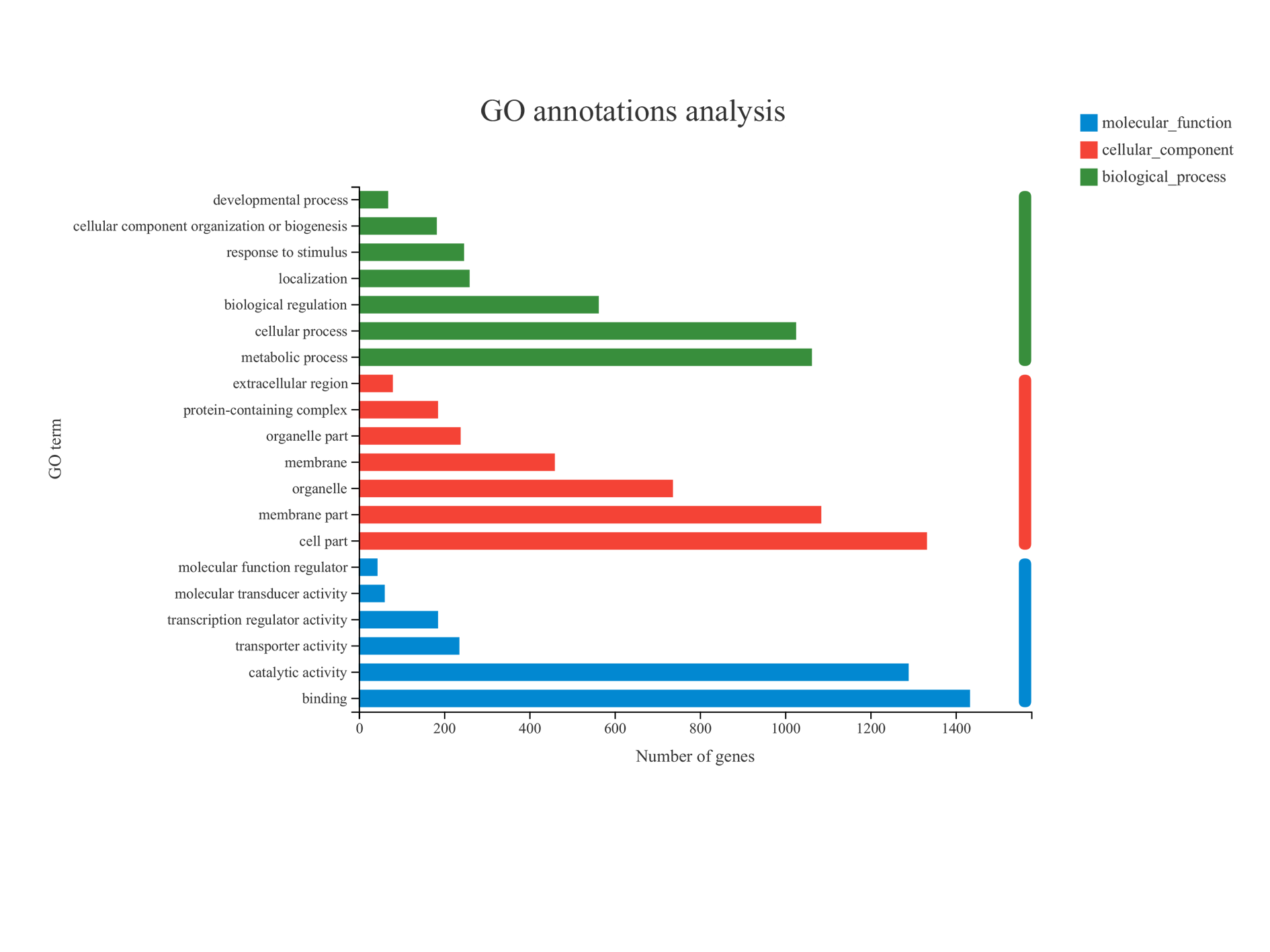

Supplement: Supplementary Figure 3 — GO functional annotation analysis of DEGs. [file Image_3.tif]
